# Impact of histological diagnosis on the treatment of atypical brainstem lesions

**DOI:** 10.1038/s41598-020-68063-6

**Published:** 2020-07-06

**Authors:** Marcos Dellaretti, Breno Bezerra Arruda Câmara, Pedro Henrique Piauilino Benvindo Ferreira, José Batista da Silva Júnior, Rosa Maria Esteves Arantes

**Affiliations:** 1 Department of Neurosurgery, Hospital Santa Casa de Misericórdia de Belo Horizonte, Minas Gerais, Av. Francisco Sales, 1111, Belo Horizonte, 30150-221 Brazil; 2 Department of General Pathology, Institute of Biological Sciences, Federal University, Minas Gerais, Av. Pres. Antônio Carlos, 6627, Belo Horizonte, 31270-901 Brazil

**Keywords:** CNS cancer, CNS cancer, Surgical oncology

## Abstract

For atypical brainstem lesions, histological diagnosis can have an impact on treatment, especially in cases where diffuse glioma is not found. Since radiotherapy is the only therapeutic modality that has shown clinical and radiographic improvement in patients with diffuse glioma, the misdiagnosis of diffuse glioma can have drastic consequences, particularly in patients with nontumorous lesions. Thus, the purpose of this study was to evaluate the impact of histological diagnosis on the treatment of atypical brainstem lesions. This was a retrospective study of 31 patients who underwent biopsy of atypical brainstem lesions. The procedures were performed between January 2008 and December 2018 at the Life Center Hospital and Santa Casa de Belo Horizonte, MG, Brazil. A diagnosis was obtained in 26 (83.9%) cases. Three patients presented complications: one presented bleeding with no clinical repercussions and two showed worsening of neurological deficit, only one of which was definitive. No mortality occurred due to the procedure. The histological diagnosis was diffuse glioma in seven cases (22.6%) and not diffuse glioma in 19 cases (61.3%). Thus, the histological diagnosis had an impact on the treatment of 19 patients (treatment impact rate: 61.3%). The histological diagnosis of intrinsic brainstem lesions is a safe, efficient procedure with a high diagnosis rate, and as such, it should be considered in the management of atypical lesions.

## Introduction

The treatment of patients with brainstem lesions, which account for only 1.6% of central nervous system tumors, is complex and controversial, particularly in cases in which surgical resection is not indicated^1,2^.

The diagnosis of intrinsic brainstem tumors is based on imaging studies and clinical history, particularly in cases of “typical” diffuse pontine lesions, due to the high frequency of diffuse gliomas in such cases^3^.

The imaging characteristics that are considered typical of diffuse pontine gliomas are as follows: (a) an intrinsic and central tumor involving more than 50% of the axial diameter of the pons; (b) poorly defined margins; (c) T1 hypointensity; (d) T2 hyperintensity; (e) heterogeneous contrast uptake, if any; and (f) the absence of cystic or exophytic components^4,5^.

Based on these radiographic parameters, a patient presenting with a clinical syndrome that is consistent with typical diffuse glioma is submitted to radiotherapeutic treatment without histological confirmation^6^. In contrast, a patient presenting with an image considered to be “atypical” requires histological confirmation of the diagnosis^7–9^. For these lesions, the image shows the following characteristics: (a) T2 hyposignal; (b) diffusion restriction; (c) enhanced prominent contrast^10^; and (d) lesions outside the limits of the pons^11^.

The differential diagnosis of these lesions ranges from nontumoral lesions, such as ischemia, demyelinating disease, vascular malformation, abscess, radionecrosis, tuberculoma and granuloma, to benign or malignant tumors, such as epidermoid cysts, metastatic tumors, and lymphoma, among others^12–18^.

Thus, the histological diagnosis of atypical lesions may have an impact on treatment, particularly in cases in which diffuse glioma is not found. Since radiotherapy is the only therapeutic modality that has shown clinical and radiographic improvement in patients with diffuse gliomas, the misdiagnosis of diffuse glioma can have drastic consequences, particularly in patients with nontumorous lesions^11,19,20^.

The purpose of this study was to evaluate the impact of histological diagnosis on the treatment of atypical intrinsic brainstem lesions.

## Methods

### Sample

From January 2008 to December 2018, 103 patients with brainstem tumors were admitted to the Life Center Hospital and Santa Casa de Belo Horizonte, MG, Brazil, of which 31 were considered to have atypical tumors. All patients with atypical tumors underwent biopsy. This study analyzed the records of 31 patients who underwent biopsy of atypical brainstem lesions.

This study was approved by the *Santa Casa de Belo Horizonte* Ethics Committee (049/2011). The need to obtain informed consent from the patients was waived by the ethics committee, which approved this study’s protocols.

The patients evaluated included 18 (58%) men and 13 (42%) women. The mean age of the patients was 29.4 years (standard deviation (SD) 95% CI: 21.2 to 37.6 years), ranging from 3 to 75 years. Fourteen patients were children (< 18 years old), and 17 were adults (≥ 18 years).

The mean time between the onset of symptoms and biopsy was 68.6 days (SD 95% CI: 33.8 to 103.3 days), ranging from 1 to 360 days (Table 1).

**Table 1:** Characteristics.

Characteristic	Sample
Age (mean, years)	29.4
**Sex**	
Male Female	18 13
**Location**	
Pons Midbrain Medulla oblongata	20 9 2
**Biopsy**	
Stereotaxic Craniotomy	30 1
**Anesthesia**	
Local General	17 14

The symptoms included hemiparesis in 11 patients, gait ataxia in 7, facial paresis in 5, nerve III paresis in 5, diplopia in 2, vertigo in 2, dysphagia in 1, tetraparesis in 1, dysphonia in 1 and signs of intracranial hypertension in 1.

The location of the lesion in the brainstem was the pons in 20 (64.5%) patients, the midbrain in 9 (30.0%) patients and the medulla oblongata in 2 (6.5%) patients.

### Surgical technique

The transfrontal approach was chosen in most cases, except in one case that had a middle pontine lesion with infiltration of the middle cerebellar peduncle, for which a suboccipital approach was preferable. Stereotactic biopsy was performed in 30 patients: a transfrontal approach was used in 29 (93.6%) patients and a suboccipital approach was used in 1 (3.2%) patient.

For one patient (3.2%), the biopsy was performed by craniotomy. This patient had an intrinsic pontine lesion with an extrinsic portion in the middle cerebellar peduncle.

In the children patients, stereotaxic biopsy was performed under general anesthesia, and in the adult patients, it was performed under local anesthesia and sedation. Thus, local anesthesia and sedation were used in 17 (54.8%) patients, and general anesthesia was used in 14 (45.2%) patients. This procedure was guided by tomography in 25 patients and magnetic resonance in 5.

The procedure was considered successful in all cases where a diagnosis was obtained. All biopsy specimens were formalin fixed and analyzed after staining with hematoxylin & eosin (HE), Masson trichrome and immunostaining. The biopsy findings were correlated with the clinical status of the patients.

### Atypical brainstem lesions

For these lesions, the image usually shows the following characteristics: (a) T2 hyposignal; (b) diffusion restriction; (c) enhanced prominent contrast; (d) lesions outside the limits of the pons; and (e) cystic components^3,4,11^. Thus, 11 patients showed enhanced prominent contrast, 5 showed diffusion restriction, 11 showed lesions outside the limits of the pons and 5 showed cystic components.

### Impact of histological diagnosis

The histological diagnosis was considered to have had an impact on treatment in cases where diffuse glioma was not found.

### Lesion classification

The lesions were classified into the following four groups: (I) diffuse non-contrast-enhanced lesion, magnetic resonance imaging (MRI) showing hypointense diffuse lesion in T1, non-contrast-enhanced in T1 after contrast injection, and diffuse in T2; (II) diffuse contrast-enhanced lesion, MRI showing hypointense diffuse lesion in T1, contrast-enhanced in T1 after contrast injection, and diffuse in T2; (III) non-contrast-enhanced focal lesion, MRI showing hypointense focal lesion in T1, non-contrast-enhanced in T1 after contrast injection, and focal in T2; and (IV) contrast-enhanced focal lesion, MRI showing hypointense focal lesion in T1, contrast-enhanced in T1 after contrast injection, and focal in T2^21^.

### Data analysis and statistical analysis

Data analysis was performed using MedCalc 9.3.0.9 software. We calculated the treatment impact rate, together with the rates of diagnosis, morbidity and mortality related to the biopsy for histological diagnosis. Age, contrast uptake and resonance characteristics were analyzed to identify whether they were associated with the histological diagnosis.

The x^2^ test was used to evaluate categorical variables, and analysis of variance was used to compare mean values. A P value < 0.05 was considered statistically significant in all analyses.

## Results

### Image characteristics

Evaluation of the T1 weighted images of MRI showed that 30 (96.8%) lesions were hypointense and 1 (3.2%) was hypointense and hyperintense. In the contrasted T1 weighted images, 15 (48.4%) showed heterogeneous contrast enhancement, 11 (35.5%) showed homogeneous contrast enhancement and 5 (16.1%) showed no contrast enhancement. In the T2 weighted images, 3 were hypointense, 23 were hyperintense and 4 were hypointense and hyperintense.

Based on the characteristics of the MRI images, the lesions were diffuse contrast-enhanced (Type II) in 15 (48.4%) cases, focal contrast-enhanced (Type IV) in 11 (35.5%) and diffuse non-contrast-enhanced (Type I) in 5 (13.3%). There were no focal noncontrast-enhanced (Type III) lesions.

### Success rate and complications

A diagnosis was obtained in 26 (83.9%) of 31 cases; thus, no diagnosis was obtained in 5 (16.1%). Three patients presented complications: one presented bleeding with no clinical repercussions and two showed worsening of neurological deficit, only one (3.2%) of which was definitive. No mortality occurred due to the procedure.

### Impact of histological diagnosis

Histological diagnoses of diffuse glioma were obtained in 7 cases (22.6%) (3 cases of diffuse high-grade glioma and 4 of diffuse low-grade glioma), while in 19 (61.3%), the diagnosis did not indicate diffuse glioma, and in 5 (16.1%) cases, the biopsy was inconclusive. Thus, the histological diagnosis had an impact on the treatment of 19 patients (treatment impact rate: 61.3%).

The H3K27M mutation was present in six of seven cases of diffuse brainstem glioma.

We verified a broad range of lesions in patients who had no histological diagnosis of diffuse glioma: pilocytic astrocytoma in 5 (16.1%) cases, B-cell lymphoma in 4 (12.9%), inflammatory infiltrate in 2 (6.5%), germinoma in 2 (6.5%), ependymoma in 1 (3.2%), ganglioglioma in 1 (3.2%), toxoplasmosis in 1 (3.2%), metastasis in 1 (3.2%), actinic lesion in 1 (3.2%) and abscess in 1 (3.2%) (Table 2).

**Table 2:** Location and Histological Diagnosis.

Location	Histological diagnosis (N)
Midbrain	2 Germinoma 2 B-cell lymphoma 1 Diffuse high-grade glioma 1 Ganglioglioma 1 Toxoplasmosis 1 Inflamatory infiltrate 1 Inconclusive
Pons	5 Pilocytic astrocytoma 3 Diffuse high-grade glioma 2 Diffuse low-grade glioma 2 B-cell lymphoma 1 Ependymoma 1 Metastasis 1 Actinic lesion 1 Abscess 1 Inflammatory infiltrate 3 Inconclusive
Medulla oblongata	1 Diffuse low-grade glioma 1 Inconclusive

Of the patients with pilocytic astrocytoma, two underwent craniotomy. The patients with lymphoma underwent chemotherapy, and three of them also received radiotherapy. The patient with metastasis underwent radiosurgery. The patients with inflammation underwent corticosteroid pulse therapy and imaging follow-up. The patient with an abscess was treated with antibiotics and toxoplasmosis to treat neurotoxoplasmosis. The patients with ependymoma and germinoma underwent stereotactic radiotherapy. Three patients with pilocytic astrocytoma and one patient with ganglioglioma did not undergo any intervention and were followed by imaging.

Regarding the patients in which the biopsy did not have success, these five patients were followed up by MRI. Of these, three had progression of the lesion, and one was submitted to biopsy by endoscopy, which revealed germinoma. Two patients underwent craniotomy, of which one was revealed to have lymphoma and the other was diffuse brainstem glioma. Two patients had no progression of the lesions and maintained only the MRI follow-up.

### Factors associated with the impact of histological diagnosis

Regarding age, the mean age was 22.2 years in patients with a diagnosis of diffuse glioma and 30.5 years in those with no diagnosis of diffuse glioma. This difference was not statistically significant (p = 0.430). In addition, there was no difference in the impact of biopsy between children and adults, since the diagnosis of diffuse glioma was found in four children and three adults (p > 0.05) (Table 3).

**Table 3:** Age and histological diagnosis.

Age	Histological diagnosis (N)
Children	2 Diffuse high-grade glioma- 2 Diffuse low-grade glioma 5 Pilocytic astrocytoma 1 Actinic lesion 1 Ependymoma 1 Germinoma 1 Ganglioglioma 1 Inconclusive
Adults	2 Diffuse low-grade glioma 1 Diffuse high-grade glioma 4 B-cell lymphoma 2 Inflammatory infiltrate 1 Metastasis 1 Toxoplasmosis 1 Germinoma 1 Abscess 4 Inconclusive

*N* number.

Considering only patients for whom the procedure was successful in obtaining a diagnosis (26 patients), of the 19 patients in which diffuse glioma was not diagnosed, 17 presented contrast-enhancing lesions, while among the 7 patients in which diffuse glioma was diagnosed, 6 presented contrast-enhancing lesions (p = 0.67).

Regarding the MRI parameters, we observed that contrast-enhancing focal or type III lesions showed a lower frequency of diffuse glioma diagnosis than the other categories. Of these patients, for the 8 patients in which a diagnosis was obtained, none had diffuse glioma, while among the other 18 patients, diffuse glioma was verified in 7. This difference was not statistically significant (p = 0.062).

### Illustrative case

**Figure 1:**
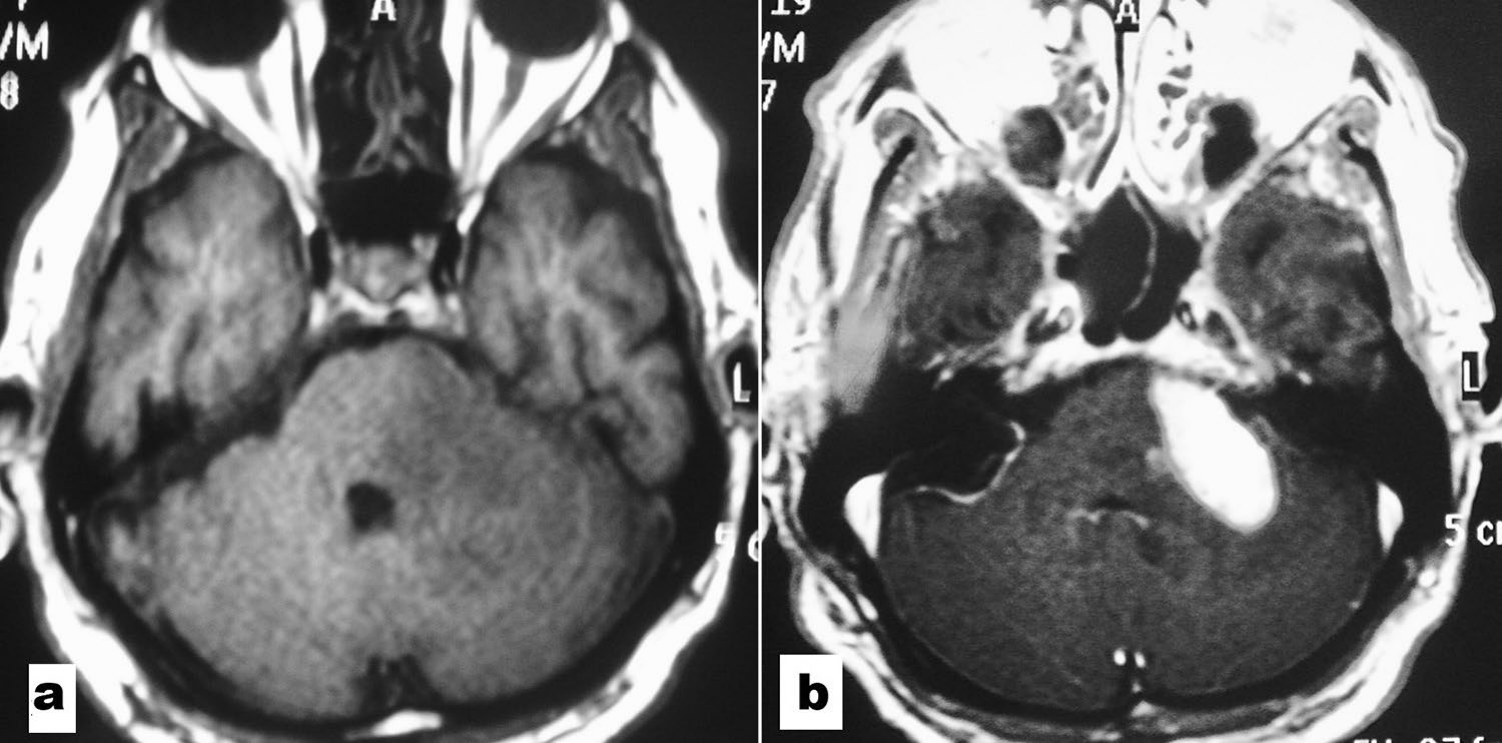
Magnetic resonance imaging appearance of an atypical brainstem tumor. **(a)** T1-weighted axial image without and **(b)** with gadolinium demonstrates an enhanced prominent contrast and lesion outside the limits of the pons.

**Figure 2:**
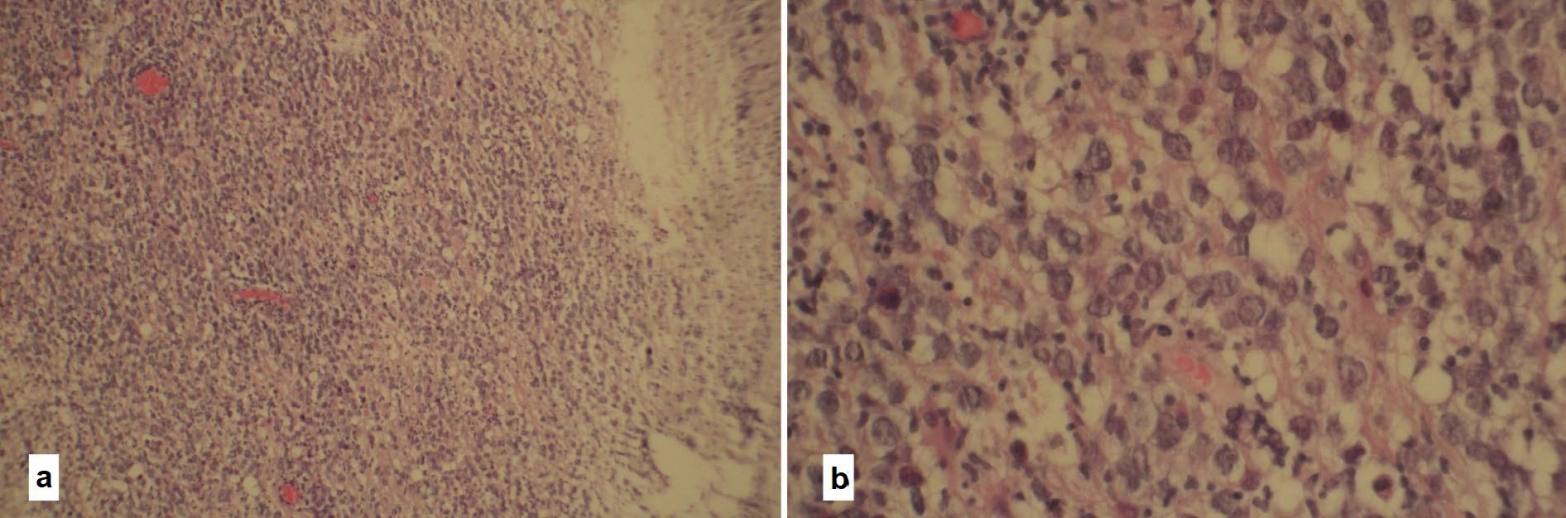
Hematoxylin and eosin (H&E): (**a)** shows increased cellularity with irregular nuclei, scarce cytoplasm and perivascular accumulation of cells. (**b** shows scattered binucleated and multinucleated cells.

**Figure 3:**
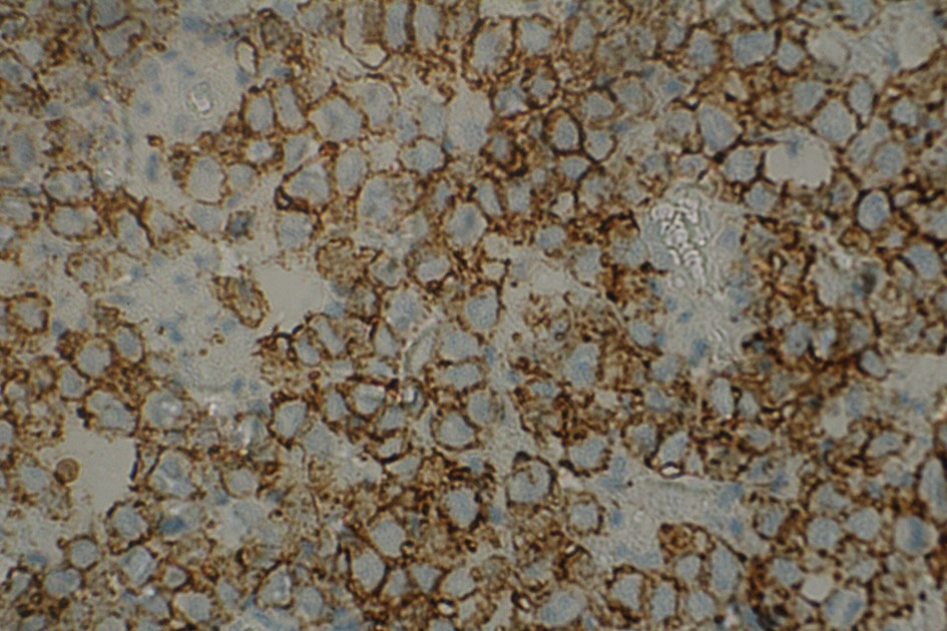
Immunohistochemistry. The histological field (×400 objective) shows strong positivity for CD20.

A 72-year-old patient presented with gait instability. Neurologic exam showed dysmetria. MRI scans were performed, and imaging revealed an atypical brainstem tumor (enhanced prominent contrast and lesion outside the limits of the pons) of the pons and left middle cerebellar peduncle (Fig. 1). A stereotaxic biopsy was performed by the frontal approach. The histopathological examination revealed lymphoma by H&E staining (Fig. 2a, b) and strong immunopositivity for CD20 (Fig. 3).

## Discussion

In this study, the biopsy diagnosis rate was 83.9%, and permanent morbidity occurred in only one patient. The literature shows a diagnostic biopsy rate in brainstem lesions ranging from 87 to 100%^22^.

Regarding morbidity, data from the literature show discrepancies. Ogiwara et al. (2013)^23^ reported a case of worsening of neurological deficit; however, since their series only involved seven cases, this represents a morbidity rate of 14.2%. In their study involving 30 patients submitted to stereotaxic biopsy, Massager et al. (2000)^24^ verified only two complications, which was 6.7% of the cases studied. In a systematic review, Hamisch et al.^25^ reported a morbidity of 6.7%, with 0.6% definitive morbidity and 0.6% mortality. In a review by Kickingereder et al.^22^ of 1,480 cases, the permanent morbidity rate was 1.7%, and mortality was 0.9%.

A diagnosis of diffuse glioma was only verified in seven cases (22.6%), which is a low frequency compared with that in other studies. This finding can be explained by our evaluation of only those lesions considered “atypical”. In the majority of series of brainstem lesion biopsies found in the literature, this subdivision is not considered ^15,21,22,26–30^.

In their review, Samadani et al.^26^ analyzed the diagnoses of 203 adults with brainstem lesions, and a histological diagnosis of diffuse glioma was found in 56% of cases. León et al.^27^ evaluated 50 patients from six months to 15 years old with a cranial CT suggestive of diffuse pontine glioma. A histological diagnosis of diffuse glioma was verified in 86% of the cases. Pincus et al.^29^ published ten cases of children submitted to stereotaxic biopsy, and only three cases were diagnosed with pathologies different from diffuse glioma; one was a demyelinating lesion, and the other two were tumors of nonglial origin (central neurocytoma and medulloblastoma). Pirotte et al.^28^ reported a series of 20 children with diffuse brainstem lesions who underwent biopsy using 18F-fluorodeoxyglucose (FDG) PET. In only five cases, the diagnosis was not diffuse glioma (two PNETs, one teratoma and two germinomas).

Regarding the impact of the histological diagnosis on the treatment, in our study, 19 cases presented a diagnosis different from diffuse glioma, that is, histology had an impact on treatment in 61.3% of cases.

In these patients, a wide variety of treatments were performed, ranging from imaging follow-up to tumor resection, chemotherapy, radiosurgery and others. Some studies have also indicated that histological diagnosis has an impact on cases in which diffuse gliomas are not present^15^.

Rachinger et al.^15^ submitted a sample of 46 adults with brainstem lesions to stereotactic biopsy and verified six nontumor lesions and 13 nonglial tumors (seven metastases and five lymphomas). Thus, the biopsy had an impact on the treatment in 19 (41.3%) of the 46 patients.

Although no significant difference was observed in the focal and contrast-enhancing lesions, the impact of the histological diagnosis was greater in this group of lesions since the diagnosis of non-diffuse glioma was made in all cases. Other studies show similar results, including one that correlated the histological and imaging findings, demonstrating that the biopsy had an impact on treatment, particularly for focal lesions, in which the diagnosis of diffuse glioma was obtained in only 35.7% of cases^21,30^.

Thus, obtaining a histological diagnosis in atypical lesions has an important impact on treatment due to the broad range of pathologies found and the lower frequency of diffuse gliomas.

### Diffuse pontine gliomas and research progress

In most studies, treatment decisions for “typical” diffuse pontine glioma are based on MRI features alone and do not include histopathological diagnosis. Several authors regard biopsy procedures for intrinsic brainstem tumors as being too dangerous and consider imaging methods as sufficiently reliable^30^.

Recently, the increased safety and feasibility of stereotactic biopsy, together with research developments of clinical significance, command a reexamination of this stance. Current data show that stereotactic biopsy can be performed with minimal morbidity and mortality and is associated with a high pathological and molecular diagnostic yield, making a strong case for carefully executed biopsy in patients with suspected diffuse pontine glioma^31^.

In addition, increased tissue acquisition has allowed for an improved understanding of the molecular biology of diffuse pontine glioma and its increasing number of known variants, opening the door to new prospects in individualized medicine and targeted trials^30,31^.

For example, epigenetic studies have identified oncogenic transcription targets, including CDK7 blockade and BRD4 inhibition^32^. Antitumor activity has been demonstrated by inhibiting K27 demethylase JMJD3 via GSKJ4, showing promise in targeting defective transcription mechanisms in diffuse brainstem glioma^26^.

### Study limitations

This study is subject to bias for two reasons: it is a retrospective study of medical records, and a single surgeon performed the series of biopsies. However, despite these limitations, to our knowledge, this is the first study to only consider the MR diagnosis of atypical intrinsic brainstem lesions in the literature, providing important information for brainstem lesion management.

## Conclusion

The histological diagnosis of intrinsic brainstem lesions is a safe, efficient procedure with a high diagnosis rate, and as such, it should be considered in the management of patients with atypical lesions who are not candidates for surgical resection.
